# Endometrial Microbes, Systemic Inflammatory Markers, and In Vitro Fertilization Outcomes Among Infertile Women: A Cross-Sectional Study

**DOI:** 10.7759/cureus.117803

**Published:** 2026-10-12

**Authors:** Kumudini Panigrahi, Mousumi Acharya, Jyoti Prakash Sahoo, A Raj Kumar Patro, Dipti Pattnaik

**Affiliations:** 1 Microbiology, Kalinga Institute of Medical Sciences, Bhubaneswar, IND; 2 Obstetrics and Gynaecology, Kalinga Institute of Medical Sciences, Bhubaneswar, IND; 3 Pharmacology, Kalinga Institute of Medical Sciences, Bhubaneswar, IND

**Keywords:** anti-inflammatory cytokine, endometrial microbiota, induced abortion, in vitro fertilization (ivf), primary and secondary infertility, pro-inflammatory cytokines, recurrent implantation failures, recurrent pregnancy loss (rpl), social stigma, tubal infertility

## Abstract

Background and objectives: Infertility among couples of the reproductive age group is a global healthcare concern. Imbalance in the neuro-immune-endocrine network leads to altered pro- and anti-inflammatory markers and endometrial dysbiosis. These two factors collectively cause infertility, despite the advancements in in vitro fertilization (IVF). Indian studies exploring this aspect are scarce. Therefore, we planned to evaluate endometrial microbes, interleukin-10 (IL-10), and IL-17 among infertile women seeking IVF.

Methods: This cross-sectional study was conducted from January 2025 to June 2026 at Kalinga Institute of Medical Sciences (KIMS), Bhubaneswar, India. Infertile women aged 31-40 years seeking treatment with IVF were included in this study. Those with active vaginal or cervical infection, any autoimmune disease, or use of antimicrobials, corticosteroids, or immunosuppressive agents were excluded. The endometrial and blood samples were obtained in the peri-implantation window. Cultivable isolates were identified using matrix-assisted laser desorption ionization-time-of-flight mass spectrometry (MALDI-TOF MS; BioMérieux, France). Serum IL-10 and IL-17 levels were detected using enzyme-linked immunosorbent assay (ELISA). We used R software (version 4.6.2) for data analysis.

Results: Our study population comprised 50 infertile women. The study population’s median age was 37 (35-38) years. The median body mass index (BMI) of the study participants was 26.75 (24.20-30.80) kg/m^2^. Of the study participants, 32 (64%) had primary infertility. Of the 50, 17 (34%) had a tubal cause of infertility. *Lactobacillus *spp. (23, 46%) was the most common microbe in the endometrial cultures, followed by *Enterococcus faecalis* (5, 10%) and *Klebsiella pneumoniae* (5, 10%). A total of 20 (40%) participants became pregnant following IVF treatment. The remaining 30 (60%) had IVF failure. The median IL-10 values for participants with IVF failure and success were 14.93 (13.87-15.96) pg/mL and 35.60 (32.53-39.94) pg/mL, respectively (p < 0.001). The corresponding median IL-17 values were 40.02 (34.84-44.29) pg/mL and 23.57 (21.06-24.36) pg/mL (p < 0.001). The 20 participants who were pregnant with IVF had lower IL-17 levels and higher IL-10 levels. The differences were statistically significant. The IVF success rates were seen among those aged 31-35 years (8 of 15, 53.3%), secondary infertility (9 of 18, 50%), those with *Lactobacillus *spp. (14 of 23, 60.9%), and those with previous abortion (13 of 28, 46.4%).

Conclusion: The commonest microbe in the endometrial cultures was *Lactobacillus *spp. Of the 20 participants with successful IVF, two-thirds had positive endometrial cultures for *Lactobacillus *spp. We also found that higher IVF success rates were seen among those with secondary infertility and aged between 31 and 35 years. Participants with IVF failure had higher IL-17 levels and lower IL-10 levels.

## Introduction

Infertility is regarded as a disease of the couple. It is defined as the inability to achieve a clinical pregnancy after 12 consecutive months of regular unprotected sexual intercourse (at least twice weekly) [1]. It leads to psychological, physical, and mental stress. Moreover, its social stigma can cripple marital harmony and family peace [2-4]. In the last three decades, the global incidence of female infertility has skyrocketed by 84.4% [2]. The worldwide cases of female infertility have increased from 59.7 million in 1990 to 110.1 million in 2021, with an increasing trend in the age-standardized prevalence rate [2]. Around one-tenth of couples from the reproductive age group face infertility issues, and this burden is significant among females aged 35-49 years [3,4]. In India, according to the National Family Health Survey-4 (NFHS-4) in 2015-2016, the infertility rate was approximately 7% [5,6]. According to the NFHS-5 reports in 2019-2021, the infertility rate has surged to 12% [5,6]. Regardless of the impacts of infertility in India, access to infertility care remains hindered owing to a lack of obvious symptoms and unawareness of the gravity of this stigmatizing issue [5].

Primary infertility is defined as the inability to conceive despite cohabitation for 12 consecutive months [7,8]. If the female has conceived previously, then it is regarded as secondary infertility [7,8]. In developing countries like India, primary infertility is more serious and stigmatizing than secondary infertility [6,7]. The most common causes of female infertility are ovulatory dysfunction, tubal pathology, endometriosis, pelvic adhesions, sexually transmitted infections (STIs), obesity, alcoholism, and smoking [6]. Similarly, the most common causes of male infertility are primary testicular failure, varicocele, and abnormalities in sperm morphology and motility [9]. Induced abortions increase the likelihood of secondary infertility [6]. Implantation failure and recurrent pregnancy loss (RPL) are the major challenges for successful reproductive outcomes despite assisted reproductive technologies (ART) such as in vitro fertilization (IVF) [5,6,8]. Therefore, the evaluations must entail the following aspects: ovulatory dysfunction, spermatogenesis-related disorders, recurrent implantation failure (RIF), RPL, fetal growth anomalies, and obstetric complications [10]. Globally, RIF affects around 10%-15% of infertile women despite IVF treatment [11,12]. RIF is best defined as failure to attain a positive pregnancy following ≥3 subsequent transfers of good-quality embryos [11,13]. An exaggerated response by immunocompetent cells or inadequate tolerance at any of the abovementioned stages can lead to immunological infertility [10].

A stable endometrial immune system is essential for embryo acceptance and implantation in IVF [11]. The immune cells enter the endometrium from the maternal peripheral blood [10]. Th1, Th2, and Th17 cells promote a pro-inflammatory environment that facilitates the implantation of syncytiotrophoblast cells into the decidua [10,11]. This is counterbalanced by the anti-inflammatory response of the regulatory T cells (Tregs). Labor induction requires a pro-inflammatory environment to support uterine contractions, fetal delivery, and expulsion of the placenta [11]. The dynamic interplay of pro- and anti-inflammatory responses regulates the IVF outcomes [10,14]. The common anti-inflammatory cytokines are interleukin-10 (IL-10), IL-6, and IL-11 [11]. Similarly, common pro-inflammatory cytokines include tumor necrosis factor-α (TNF-α), interferon-γ (IFN-γ), and IL-17 [11]. Recent studies found that women with RIF have lower levels of IL-10 and higher levels of TNF-α, IFN-γ, and IL-17 [11,15]. Women with chronic endometritis have functional decline of Tregs and reduced IL-10 expression in the endometrium [16]. Immunomodulation is further exacerbated by elevated IL-17 expression [16]. Alterations in IL-10 and IL-17 concentrations and activities lead to RIF [11,16].

The favorable interaction between a viable embryo and a receptive endometrium is mandatory for embryo implantation [17]. Although the uterine cavity was earlier regarded as a sterile environment, advances in molecular techniques have found a microbial community within the endometrium [18]. A favorable endometrial microbiota is equally essential for a successful pregnancy [19]. A *Lactobacillus*-predominant microbiota is favorable for implantation and pregnancy outcomes [18,19]. Lactic acid inhibits the growth of other bacteria and maintains homeostasis without affecting inflammatory pathways [20]. However, microbial dysbiosis adversely affects embryo implantation and pregnancy maintenance [17-20]. Advances in next-generation sequencing (NGS), particularly 16S rRNA gene sequencing, have helped us understand the role of the endometrial microbiome in reproductive outcomes [18,21]. However, differences in study populations, sampling methods, and sequencing platforms contribute to inconsistent findings [21]. Hence, we designed this study to assess endometrial cultivable microbes and serum IL-10 and IL-17 levels in infertile women undergoing IVF.

## Materials and methods

Between January 2025 and June 2026, the cross-sectional study was conducted at Kalinga Institute of Medical Sciences (KIMS). Before commencing the study, we obtained ethical approval from the Institutional Ethics Committee (KIIT/KIMS/IEC/1935/2024, dated 18/12/2024). The study complied with the Declaration of Helsinki, Good Laboratory Practices, and institutional rules.

Study participants

Infertile women aged 31-40 years seeking treatment with IVF using their own oocytes were enrolled in this study. Those with active vaginal or cervical infection, hydrosalpinx evidenced by hysterosalpingography, uterine abnormalities (i.e., unicornuate, bicornuate, or septate uterus) evidenced by ultrasound or hysteroscopy, or a history of smoking or alcohol consumption were excluded. Those with any autoimmune disease and those on any antimicrobials, corticosteroids, or immunosuppressive agents were also excluded. Patients whose husbands were diagnosed with azoospermia were also excluded. We also excluded patients with undetectable values of IL-10 or IL-17. Patients enrolled in other studies were also excluded.

Study objectives

We planned this study to determine the endometrial microbiota and inflammatory markers in infertile women seeking IVF. This paper presents the cultivable endometrial microbes, IL-10, and IL-17, and their association with IVF outcomes. These findings were further analyzed based on the age group, abortion history, infertility types, and causes. The second paper will assess the non-cultivable endometrial microbiota through NGS and explore their association with IVF outcomes. Sequencing-based analysis is being undertaken to characterize the entire endometrial microbiota, including microorganisms that are undetectable by conventional culture, and the results will be reported in the second paper.

Study procedure

For each participant, complete clinical and demographic data were collected at enrollment. This included age, body mass index (BMI), duration of marriage, type of infertility, menstrual cycle characteristics, medical and surgical history, and medications. We used the Kuppuswamy classification [22] to ascertain the socioeconomic class. The endometrial and blood samples were obtained one week before and one week after the planned embryo transfer date, respectively, in the mid-secretory phase (peri-implantation window).

Endometrial Sample

During the peri-implantation window, the endometrial samples were collected, as this is the period of optimal endometrial receptivity. The participants were asked to lie down in the lithotomy position. The cervix was seen with a sterile speculum. The cervix was cleaned twice with sterile cotton swabs, which were wet with benzalkonium chloride solution to minimize contamination of the vagina. Endometrial tissue was obtained by aspiration with the Pipelle endometrial suction catheter, a minimally invasive aspiration device. The sterile Pipelle catheter was gently introduced through the cervical canal into the uterine cavity, avoiding the vulva and vaginal wall. The internal piston was removed to produce negative pressure. The catheter was gently spun 360° and drawn in and out to obtain endometrial tissue. The obtained endometrial biopsy was transferred into a sterile sample container. After collection, the samples were sent to the laboratory immediately to preserve the viability of the cultivable microflora. The endometrial tissue received in the laboratory was separated into three aliquots for Gram staining and microscopic evaluation, culture and sensitivity testing, and DNA extraction and 16S rRNA sequencing.

The endometrial samples were inoculated onto 5% sheep blood agar and chocolate agar plates and incubated at 37°C in a 5% CO₂ atmosphere for 48-72 hours. Following the incubation periods, all plates were inspected for macroscopically visible colonies. Phenotypically distinct colonies were isolated and cultured in pure culture. Definitive genus- and species-level identification of the cultivable isolates was performed using matrix-assisted laser desorption ionization-time-of-flight mass spectrometry (MALDI-TOF MS; BioMérieux, France) according to the manufacturer’s instructions.

Inflammatory Biomarkers

From each study participant, 2 mL of blood was collected in a clot activator vial, kept for 30 minutes, and allowed to clot. The samples were centrifuged at 2000 revolutions per minute (RPM) for 15 minutes. The collected serum samples were immediately stored in aliquots at -80°C for further testing to detect IL-10 and IL-17 by enzyme-linked immunosorbent assay (ELISA). Human IL-10 and IL-17 ELISA kits (ELK Biotechnology, Wuhan, China) were used to detect IL-10 and IL-17, respectively. The sensitivity and detection range of the human IL-10 ELISA kit were 3 pg/mL and 7.82-500 pg/mL, respectively. The sensitivity and detection range of the human IL-17 ELISA kit were 5.5 pg/mL and 15.63-1000 pg/mL, respectively.

Sandwich enzyme immunoassay was used to detect IL-10 and IL-17. The microtiter plates provided in the respective kits had been pre-coated with antibodies specific to human IL-10 and IL-17. Samples were added to the appropriate wells of the microtiter plate and incubated with biotin-conjugated antibodies specific to human IL-10 and IL-17. Then, avidin conjugated to horseradish peroxidase (HRP) was added to each microplate well and incubated. After 3,3’,5,5’-tetramethylbenzidine (TMB) substrate solution was added, wells containing human IL-10 and IL-17 exhibited a color change. The enzyme-substrate reaction was terminated with a sulfuric acid solution. The color change was measured spectrophotometrically at 450 ± 10 nm. The concentrations of human IL-10 and IL-17 in the samples were determined by comparing their optical density (OD) values.

Statistical analysis

Our goal was to determine the associations between the endometrial microbiota, inflammatory markers, and IVF outcomes in infertile women. Considering the pilot nature of this study and the cost of NGS assessment, we planned for around 50 participants. Hence, we employed non-probability convenience sampling for this study.

The Kolmogorov-Smirnov test assessed the normality of the data distribution. The collected data (demographics and inflammatory markers) were non-parametric. The categorical and continuous data were expressed as frequencies with proportions and medians with interquartile ranges (IQRs). The Wilcoxon rank-sum test was used to analyze the quantitative data (i.e., IL-10, IL-17, and IL-10/IL-17 ratio) based on the treatment outcome. Considering the descriptive nature of the categorical data, we did not perform the chi-square test. We used the R software (version 4.6.2) [23] for data analysis and plot generation. All the statistical tests were two-tailed. Statistical significance was explicated for p-values < 0.05.

## Results

We conducted this cross-sectional study from January 2025 to June 2026. We screened 3182 patients for this study. Of them, 623 (19.58%) had vaginal or cervical infections. A total of 419 (13.17%) patients had uterine abnormalities. Husbands of 709 (22.28%) patients had azoospermia. Overall, 1184 (37.21%) patients had already been enrolled in other studies. Moreover, 176 (5.53%) patients declined to participate. The IL-10 and IL-17 levels were undetectable in 21 (0.66%) enrolled subjects. Hence, they were excluded from the study. Data from the remaining 50 (1.57%) patients were assessed in this study. Table 1 shows their clinical and demographic parameters. Of the 50 participants, 35 (75%) were aged between 36 and 40 years. The study population’s median age was 37 (35-38) years. The median BMI of the study participants was 26.75 (24.20-30.80) kg/m². Overall, 20 (40%) participants had successful IVF and were pregnant. The remaining 30 (60%) had IVF failure. Moreover, 33 (66%) participants were working women. A total of 32 (64%) had primary infertility. Of the 50 patients, 17 (34%) had a tubal cause of infertility. For the remaining 33 (66%), the cause of infertility was unexplained.

**Table 1:** Sociodemographic and clinical traits of the study population Categorical variables were expressed as frequency and percentage. Continuous variables were expressed as median and IQR. IQR: interquartile range, BMI: body mass index, PCOS: polycystic ovarian syndrome

Parameters	Value
Total participants	50
Age (years)	37 (35-38)
31-35 years	15 (30%)
36-40 years	35 (70%)
BMI (kg/m^2^)	26.75 (24.20-30.80)
BMI categories	
Normal weight (18.5-24.9 kg/m^2^)	20 (40%)
Overweight (25.0-29.9 kg/m^2^)	15 (30%)
Obese (>30 kg/m^2^)	15 (30%)
Socioeconomic class	
Upper	26 (52%)
Upper middle	24 (48%)
Job status	
Working woman	33 (66%)
Housewife	17 (34%)
Regular periods	29 (58%)
History of abortion	28 (56%)
Comorbidities	
Type 2 diabetes mellitus	21 (42%)
Hypothyroidism	30 (60%)
PCOS	23 (46%)
Type of infertility	
Primary	32 (64%)
Secondary	18 (36%)
Cause of infertility	
Tubal	17 (34%)
Unexplained	33 (66%)
IVF outcomes	
Success	20 (40%)
Failure	30 (60%)

Figure 1 illustrates the distribution of the study participants using a Venn diagram. We used the following parameters for grouping: regular period (present or absent), history of abortion (present or absent), diabetes (present or absent), hypothyroidism (present or absent), PCOS (present or absent), upper socioeconomic class (yes or no), and job holder (yes or no). A total of 6 (12%) working women from the upper socioeconomic class did not have an abortion history but had irregular periods. Overall, 4 (8%) working women from the upper socioeconomic class had only hypothyroidism. Furthermore, 3 (6%) working women from the upper socioeconomic class had an abortion history and an irregular period. In addition, 5 (10%) participants had 6 of 7 parameters, and 2 (4%) participants had all 7 parameters.

**Figure 1:**
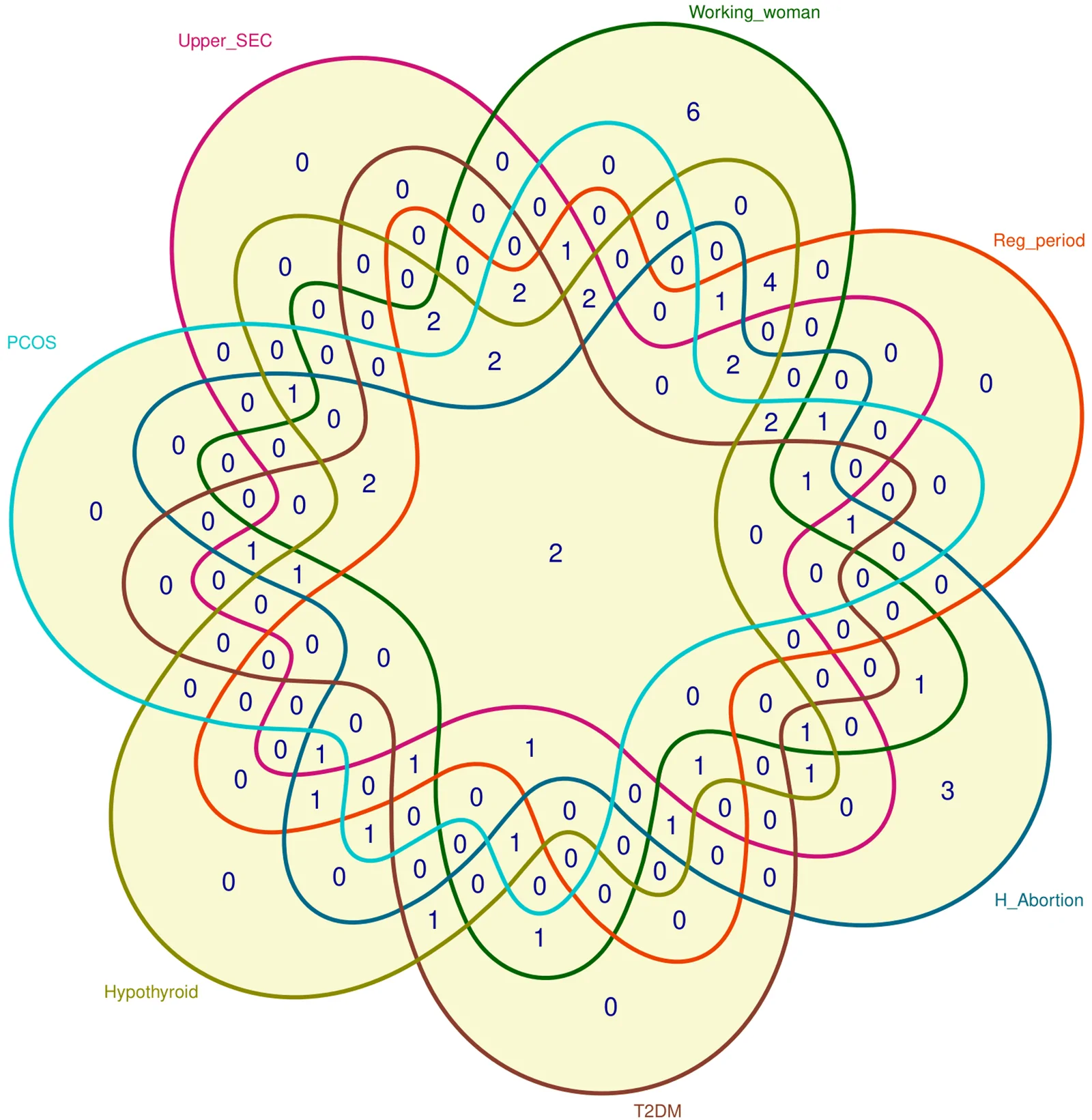
Distribution of the study participants The Venn diagram shows the study participants’ distribution according to various parameters, i.e., regular period, history of abortion, diabetes, hypothyroid states, PCOS, upper socioeconomic class, and working woman. T2DM: diabetes mellitus, Hypothyroid: hypothyroid states, PCOS: polycystic ovarian syndrome, Upper_SEC: upper socioeconomic class, Reg_period: regular period, H_abortion: history of abortion This plot was generated using R software (version 4.6.2) [24].

Figure 2 shows the types and causes of infertility among participants across age groups and BMI categories. The categorization order was age group, infertility type, BMI, and cause of infertility. The two largest blocks (six participants in each) represent the women aged 36-40 years (either normal weight or obese) with an explained cause of primary infertility. A total of 4 (8%) participants in the 36-40-year-old age group and normal BMI category had secondary infertility with an explained cause. Another 4 (8%) participants in the 31-35-year-old age group and normal BMI category had primary infertility with an explained cause.

**Figure 2:**
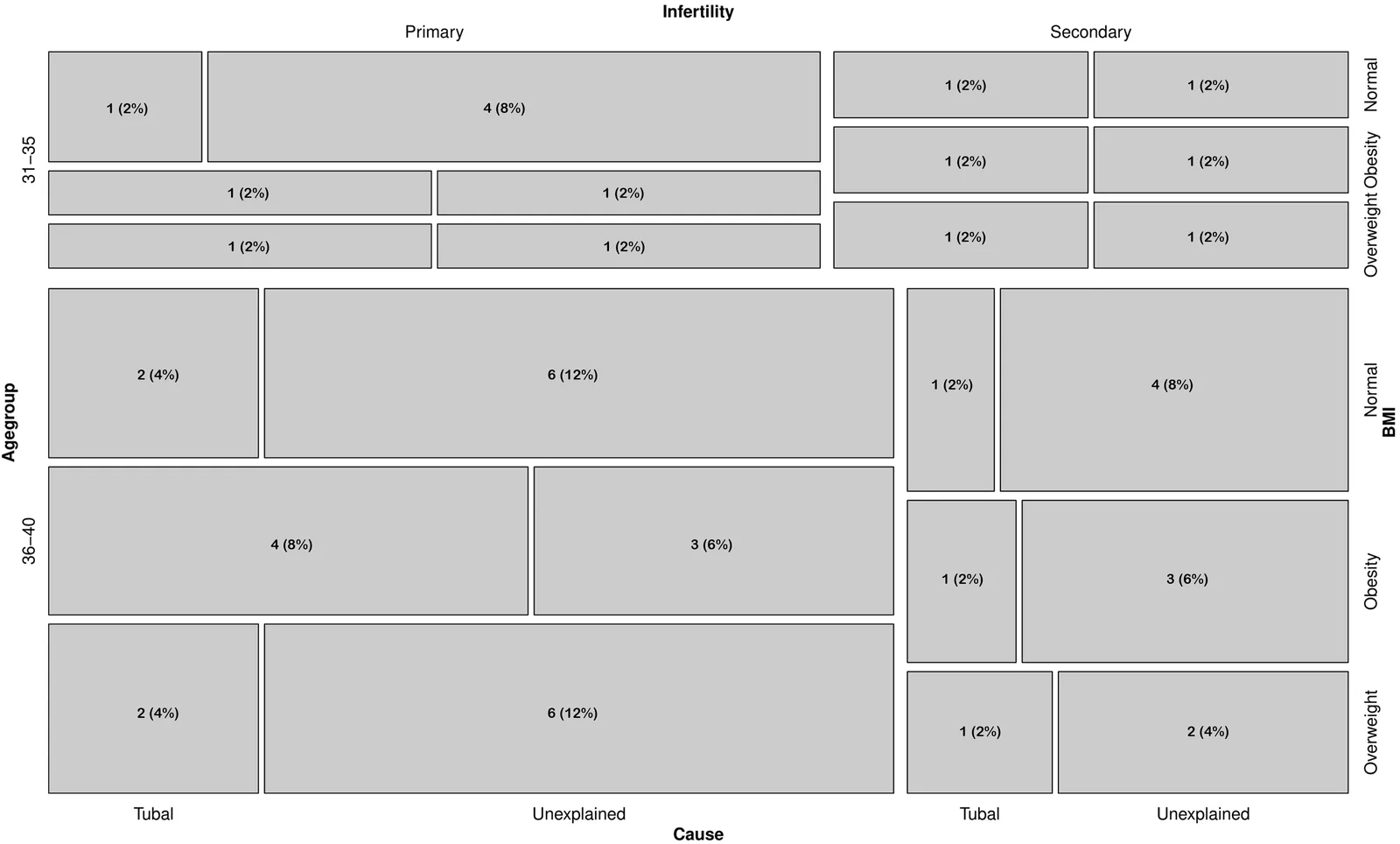
Infertility in different age groups and BMI categories The mosaic plot shows the study participants’ distribution according to various parameters, i.e., regular period, history of abortion, diabetes, hypothyroid states, PCOS, upper socioeconomic class, and working woman. BMI: body mass index, T2DM: diabetes mellitus, Hypothyroid: hypothyroid states, PCOS: polycystic ovarian syndrome, Upper_SEC: upper socioeconomic class, Reg_period: regular period, H_abortion: history of abortion This plot was generated using R software (version 4.6.2) [24].

The alluvial plot in Figure 3 illustrates the associations of IVF outcomes with age group, infertility types, history of abortion, and predominant microbe in endometrial samples. There were 35 (70%) women aged 36-40 years, 32 (64%) with primary infertility, and 28 (56%) women with a prior history of abortion. The most common microbe found in the endometrial cultures was *Lactobacillus* spp. (23, 46%), followed by *Enterococcus faecalis* (5, 10%) and *Klebsiella pneumoniae* (5, 10%). Out of 20 participants with successful IVF, 14 (70%) had *Lactobacillus* spp. in their endometrial cultures. Of those 14 participants, 11 (78.6%) had primary infertility. Of those 11 participants, 8 (72.7%) were in the 36-40-year age group. These findings revealed that higher IVF success rates were seen among those aged 31-35 years (8 of 15, 53.3%), secondary infertility (9 of 18, 50%), those with *Lactobacillus* spp. (14 of 23, 60.9%), and those with previous abortion (13 of 28, 46.4%).

**Figure 3:**
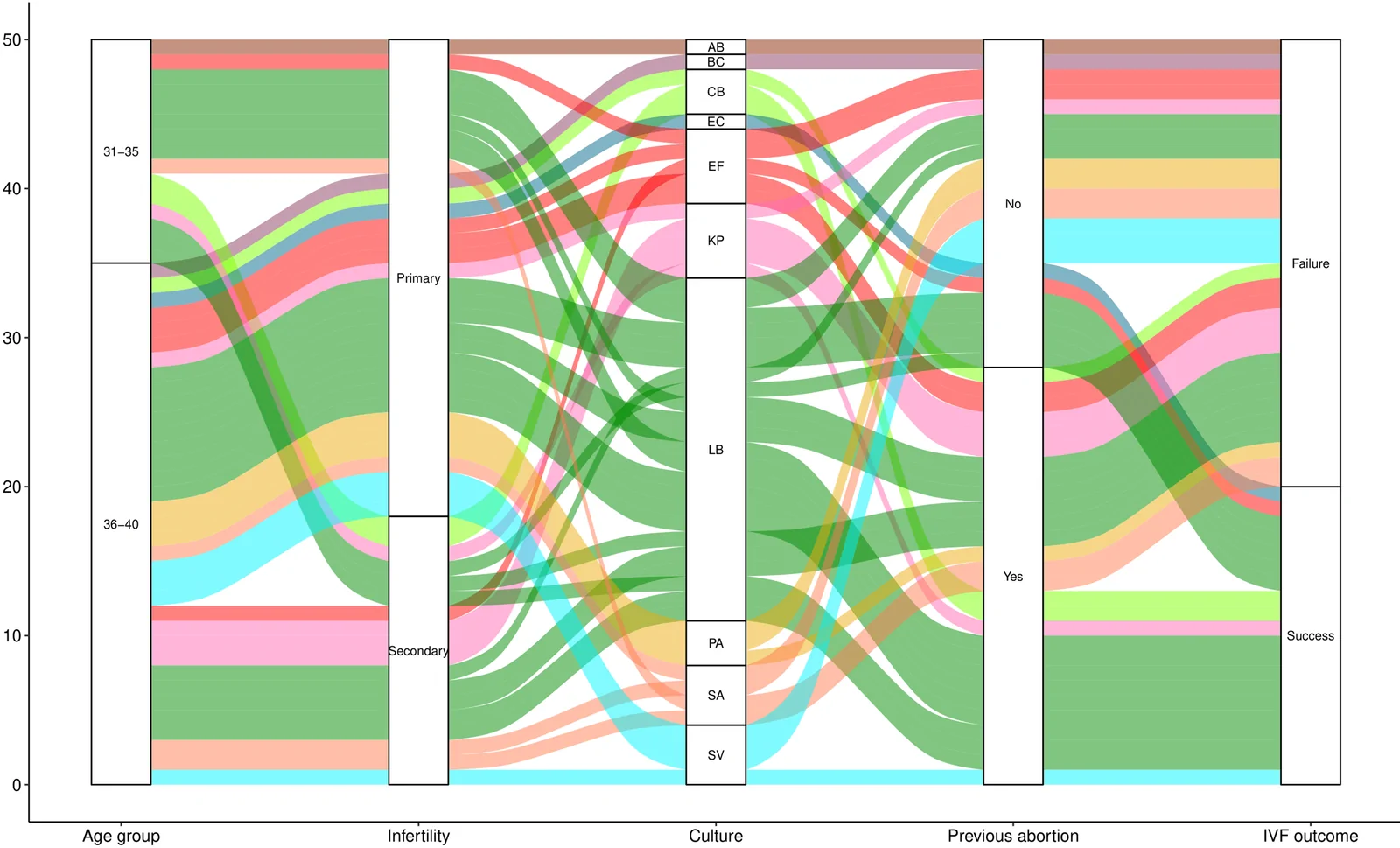
Association of IVF outcomes with various parameters The alluvial plot demonstrates the association of IVF outcomes with age group, infertility types, previous abortion history, and predominant microbes detected in endometrial sample cultures. The y-axis shows the number of participants. The microbes (i.e., AB, BC, CB, EC, EF, KP, LB, PA, SA, and SV) are shown in different colors. The bandwidths between two parameters indicate the number of participants with the same. IVF: in vitro fertilization, AB: *Acinetobacter baumannii*, BC: *Burkholderia cepacia*, CB: *Citrobacter* spp., EC: *Enterobacter cloacae*, EF: *Enterococcus faecalis*, KP: *Klebsiella pneumoniae*, LB: *Lactobacillus* spp., PA: *Pseudomonas aeruginosa*, SA: *Staphylococcus aureus*, SV: *Streptococcus viridans* This plot was generated using R software (version 4.6.2) [24].

Figure 4 illustrates the inflammatory markers (i.e., IL-10 and IL-17) of the study population. The median IL-10 of the participants was 18.02 (14.73-33.98) pg/mL. The median IL-10 values in participants (Figure 4a) with IVF failure and success were 14.93 (13.87-15.96) pg/mL and 35.60 (32.53-39.94) pg/mL, respectively (p < 0.001). The median IL-17 of the participants was 33.63 (23.86-40.77) pg/mL. The median IL-17 values for participants (Figure 4b) with IVF failure and success were 40.02 (34.84-44.29) pg/mL and 23.57 (21.06-24.36) pg/mL, respectively (p < 0.001). The median IL-10/IL-17 ratio among participants was 0.424 (0.376-1.516). The median values of the IL-10/IL-17 ratio of participants (Figure 4c) with IVF failure and success were 0.388 (0.354-0.403) and 1.545 (1.466-1.677), respectively (p < 0.001). The 20 participants who were pregnant with IVF had lower IL-17 levels and higher IL-10 levels and IL-10/IL-17 ratio. The intergroup differences were clinically and statistically significant.

**Figure 4:**
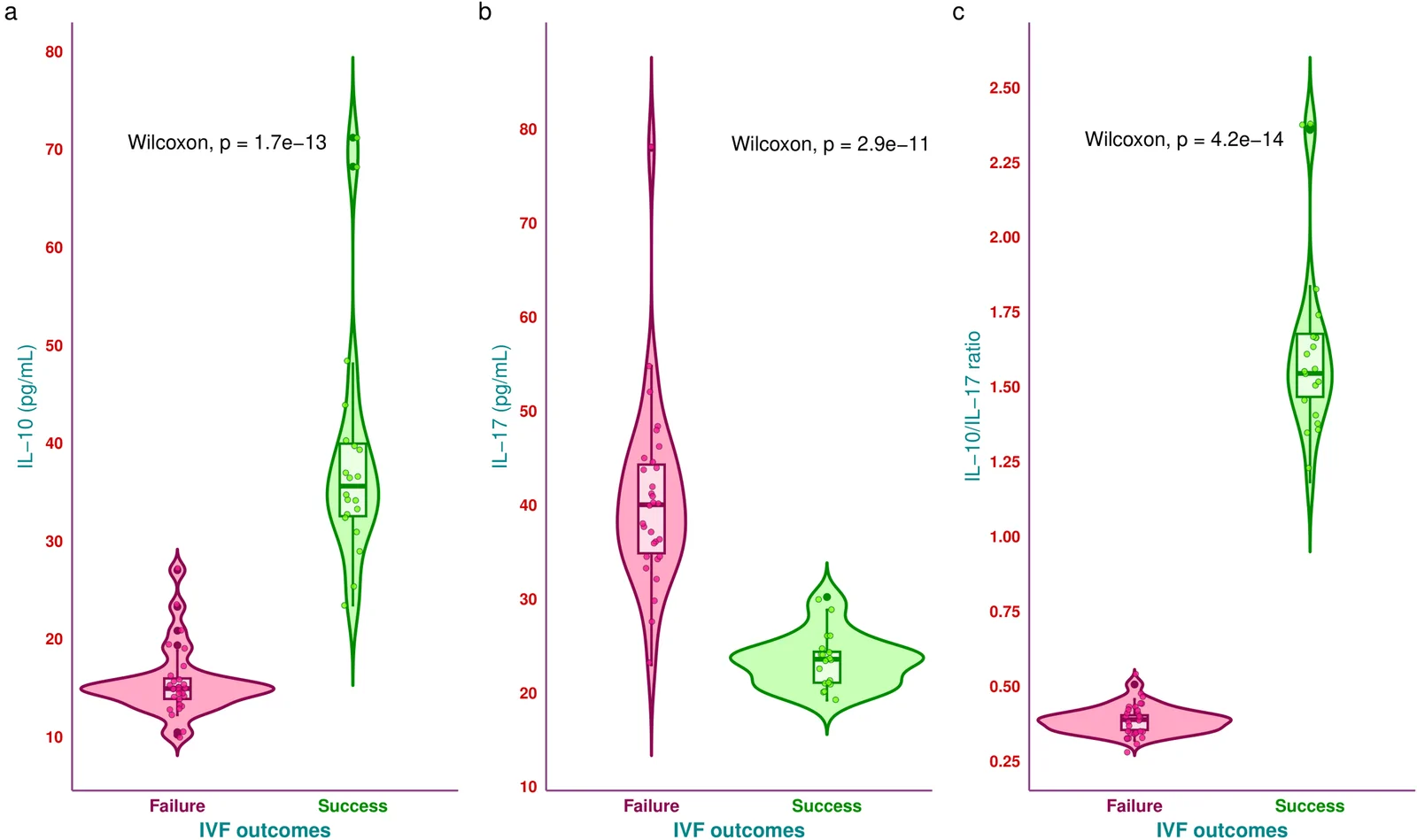
Inflammatory markers The violin, box-and-whisker, and jitter plots illustrate the inflammatory markers (i.e., IL-10, IL-17, and their ratios) of all participants (n = 50). Twenty participants became pregnant with IVF, and the remaining 30 had IVF failure. We used the Wilcoxon rank-sum test to analyze these variables. A p-value < 0.05 was considered statistically significant. IL: interleukin, IVF: in vitro fertilization This plot was generated using R software (version 4.6.2) [24].

The scatterplots in Figure 5 illustrate the distribution of inflammatory markers (i.e., IL-10 and IL-17) in the study population. Figure 5a shows that those with successful IVF had higher IL-10 and lower IL-17 values than those with failed IVF treatments. Figure 5b shows higher levels of IL-10 and IL-17 in participants aged 36-40 years, regardless of IVF outcomes. Figure 5c shows lower IL-10 values in participants with abortion history. Figure 5d shows higher IL-10 and IL-17 levels in participants with primary infertility, regardless of IVF outcomes. Figure 5c shows lower IL-10 values in participants with a tubal cause of infertility.

**Figure 5:**
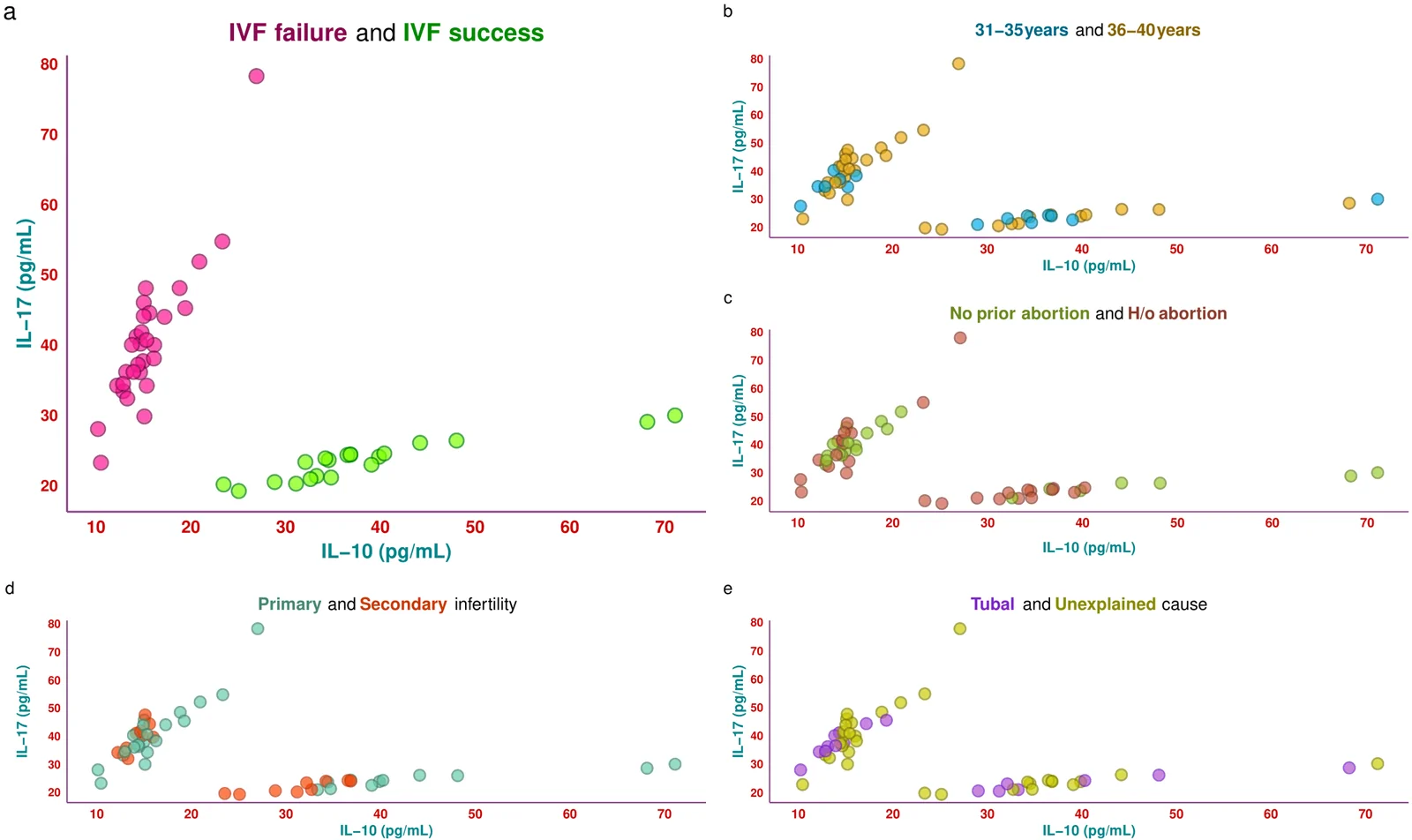
Subgroup analysis of inflammatory markers The scatterplots in panels a-e show the distribution of IL-10 and IL-17 values as per different parameters (i.e., IVF outcomes, age group, history of abortion, infertility types, and cause of infertility). IL: interleukin, IVF: in vitro fertilization This plot was generated using R software (version 4.6.2) [24].

## Discussion

In this study, we assessed both endometrial microbes and inflammatory markers in infertile women undergoing IVF treatment. We assessed the associations between IVF outcomes and endometrial microbes detected by culture and sensitivity. We also analyzed the symphony between IL-10 and IL-17 levels based on various parameters (i.e., IVF outcomes, age group, history of abortion, type, and cause of infertility). Of the 50 participants, 32 (64%) had primary infertility. A total of 17 (34%) patients had a tubal cause of infertility. Of the 50 participants, 20 (40%) achieved pregnancy with IVF treatment. *Lactobacillus* spp. was the predominant microbe in the majority (23, 46%) of the endometrial culture reports. Our findings were supported by the studies by Stoyancheva et al. [18] and Moreno et al. [19]. The 20 participants with successful IVF had reduced IL-17 levels, elevated IL-10 levels, and an elevated IL-10/IL-17 ratio. These observations concorded with the studies by Pantos et al. [11] and Wang et al. [16].

The immune system plays an important role in the reproductive system. It governs the balanced inflammatory state, which is paramount for ovulation, spermatogenesis, endometrial development, and implantation [10]. The neuro-immune-endocrine network regulates the overall function of the reproductive system [10,11,17]. Female fertility begins with proper oogenesis and the release of the oocyte during ovulation [17]. Progesterone plays an important role in successful implantation by balancing pro- and anti-inflammatory cells [24]. Suppression of the excess pro-inflammatory activity by Th1 and Th17 by Tregs after implantation is necessary for successful pregnancy and normal placental development [10]. For a successful pregnancy, the Treg/Th17 ratio must be maintained [10,24]. The functions of Tregs can be altered by an abnormal microbiome in the female reproductive tract [10].

IL-10 is a major anti-inflammatory and immunosuppressive cytokine [11,14]. Insufficient levels of IL-10 during the peri-implantation period can adversely affect embryo implantation, suggesting that dysregulation of these cytokines is an immunological factor that may lead to RIF [11,12,14]. IL-17 is a pro-inflammatory cytokine secreted explicitly by Th17 cells, which can induce a robust inflammatory response and contribute to a variety of autoimmune diseases [15,16]. These cytokines can positively regulate endometrial receptivity by promoting endometrial angiogenesis, which is crucial for embryo implantation [22,23]. In this study, we assessed the pro- and anti-inflammatory responses through the concentrations of IL-17 and IL-10, respectively. Our findings revealed that pregnant participants following IVF had higher IL-10 and lower IL-17 values than non-pregnant participants. Advanced maternal age poses an increased risk for infertility [10]. The coexistence of endometriosis and polycystic ovarian syndrome (PCOS) in infertile women disrupts the estrogen-progesterone balance and facilitates autoimmunity [10,11]. Aging and chronic immune-inflammation state cause a decline in reproductive potential [10,17].

*Lactobacillus* spp. is the most common bacterium in the female reproductive tract [18,20]. Lactic acid production by these bacteria hinders the growth of other bacteria. This ability of *Lactobacillus* spp. to reduce infection without causing inflammation might help in successful pregnancy [20,25]. Hence, bacterial dysbiosis or altered endometrial microbiota can lead to endometriosis, RIF, and RPL [18,20,25,26]. Wei et al. [27] found *Acinetobacter baumannii* and *Pseudomonas aeruginosa* in endometrial samples. Similarly, Hernandes et al. [28] found *Enterococcus* spp. and *Pseudomonas aeruginosa* in endometrial samples. Nearly half of our study participants had *Lactobacillus* spp. in their endometrial samples. However, we obtained a few cultures that were positive for *Acinetobacter baumannii* and *Pseudomonas aeruginosa*. The majority of the participants with *Lactobacillus* spp. in their endometrial samples became pregnant with IVF treatment. We are currently assessing NGS, primarily 16S rRNA gene sequencing of non-cultivable endometrial microbes from our study participants, to further explore dysbiosis and its association with IVF outcomes.

Strengths and limitations

The strengths of this study were the analyses of endometrial cultures, serum IL-10, and IL-17 among infertile women seeking IVF treatment. Our study had a few limitations as well. First, we had financial constraints owing to a lack of funding support. Hence, we could not evaluate other inflammatory markers such as TNF-α, IFN-γ, IL-6, and IL-11. We also could not reevaluate the inflammatory markers at a different time point. Second, the endometrial culture reports and the observed microbes cannot be finalized, as we did not have the NGS findings. Third, we could not evaluate endometrial dysbiosis by comparing our findings with endometrial cultures from fertile women, as this is not routinely performed at our hospital. Fourth, the sample size could not be increased due to financial burden. An increased sample size would have helped better understand the role of endometrial dysbiosis and the imbalance of inflammatory markers in IVF outcomes.

## Conclusions

In our study, the most common microbe found in the endometrial cultures was *Lactobacillus* spp. Out of 20 participants with successful IVF, two-thirds had *Lactobacillus* spp. in their endometrial cultures. Our findings revealed that higher IVF success rates were seen among those aged 31-35 years and those with secondary infertility. Participants who had successful IVF treatment had higher IL-10 and lower IL-17 levels than those with IVF failure. We recommend prospective studies with a longer study duration and a larger sample size to determine the long-term changes in inflammatory markers and endometrial dysbiosis among infertile women.
